# Identifying food marketing to teenagers: a scoping review

**DOI:** 10.1186/s12966-019-0833-2

**Published:** 2019-08-19

**Authors:** Emily Truman, Charlene Elliott

**Affiliations:** Department of Communication, Media and Film, University of Calgary, 2500 University Drive NW, Calgary, Alberta T2N 1N4 Canada

**Keywords:** Food marketing, Teenager, Adolescent, Scoping review, Food advertising, Exposure, Power, Monitor, Policy, Indicator

## Abstract

**Background:**

Teenagers are aggressively targeted by food marketing messages (primarily for unhealthy foods) and susceptible to this messaging due to developmental vulnerabilities and peer-group influence. Yet limited research exists on the exposure and power of food marketing specifically to teenage populations. Research studies often collapse “teenagers” under the umbrella of children or do not recognize the uniqueness of teen-targeted appeals. Child- and teen-targeted marketing strategies are not the same, and this study aims to advance understanding of teen-targeted food marketing by identifying the teen-specific promotion platforms, techniques and indicators detailed in existing literature.

**Methods:**

A systematic scoping review collected all available literature on food marketing/advertising with the term “teenager” or “adolescent” from nine databases, as well as Google Scholar for grey literature, and a hand search of relevant institutional websites. Included were all peer-reviewed journal articles, book chapters, and grey literature in which food marketing to youth was the central topic of the article, of any study type (i.e., original research, reviews, commentaries and reports), and including any part of the 12–17 age range.

**Results:**

The 122 articles reviewed define the scope of existing literature on food marketing to young people age 17 and under, identifying leading trends in countries studied (United States, 52%), populations identified (children and teens studied concurrently, 36%), outcomes measured (advertising exposure, 54%), study type (cross-sectional, 58%) and methods used (content analysis, 46%). The promotion *platforms* and *techniques* used by food marketers to appeal to young people (as reported in the literature) are also identified and classified. Few studies (7%) use indicators to identify teen-targeted food marketing.

**Conclusions:**

Unique treatments of teen populations are limited in food marketing literature, as is the application of clear indicators to identify and differentiate teen-targeted food marketing from child- or adult-targeted content. Given the need to better measure the presence and power of teen food marketing, this is a significant oversight in existing literature. The indicators identified will help researchers to develop more accurate strategies for researching and monitoring teen-targeted food promotion.

## Background

Regulations limiting food marketing often focus on children under age 13, despite the fact that teenagers are also susceptible to food marketing appeals [1]. Teenage vulnerability to food marketing, and especially digital promotions, stem from unique developmental factors (i.e. cognitive and emotional) [2, 3], peer-group influence [4, 5], and high levels of exposure to advertising messages [1, 6–10]. Yet research on the impact of food marketing to teenagers is limited [7, 10–12]. Teenagers, moreover, are often assessed as part of the broader category of children instead of as a unique population [11]. In fact, very few studies *define* or provide criteria to *identify* teen targeted food marketing. This scoping review examines existing studies on food marketing/advertising involving teenage populations (ages 12–17) in order to identify the scope of current research, including age ranges, outcomes, promotion platforms, techniques, and indicators. Specifically, the identification of indicators will provide a firm set of criteria for measuring the presence and power of teen-targeted food marketing.

Determining how to better identify teen-specific marketing appeals will allow for the monitoring of exposure and message content in order to inform regulatory policies on food marketing to teenagers. While many studies examine child-specific food marketing content (for example, the use of cartoon characters and ‘fun’ appeals [13–24]), little research isolates and identifies teen appeals. This is significant for teenage health since teenagers may be targeted more heavily by food marketers who are prohibited from appealing to children; it is also relevant to children, who may be attracted to teen-targeted content (WHO, 2018). Globally many countries recognize the deleterious effects of food promotion on children’s health, and have restricted or prohibited the marketing of unhealthy foods [7, 25]. Canada is also aiming to restrict food marketing to children under Bill S-228, the Child Health Protection Act, which is currently awaiting approval by the Senate [26]. However, such restrictions do not apply to teenagers, even though teenagers are influenced by marketing appeals. Indeed, teenagers’ awareness of, or cynicism towards, advertising messages does not necessarily make them more *critical* consumers or less persuaded by advertising [7, 11]. In light of the need for expanded knowledge around teen food marketing, this review is guided by the following research questions: 1) how do existing studies identify teenage populations (i.e., age range, collapsed into child populations, etc.)?; 2) how is teen-targeted food marketing identified and measured?; 3) what promotional platforms and techniques are examined?; and 4) what criteria or ‘indicators’ are used to isolate teen-specific appeals?

## Methods

### Literature search

**Fig. 1:**
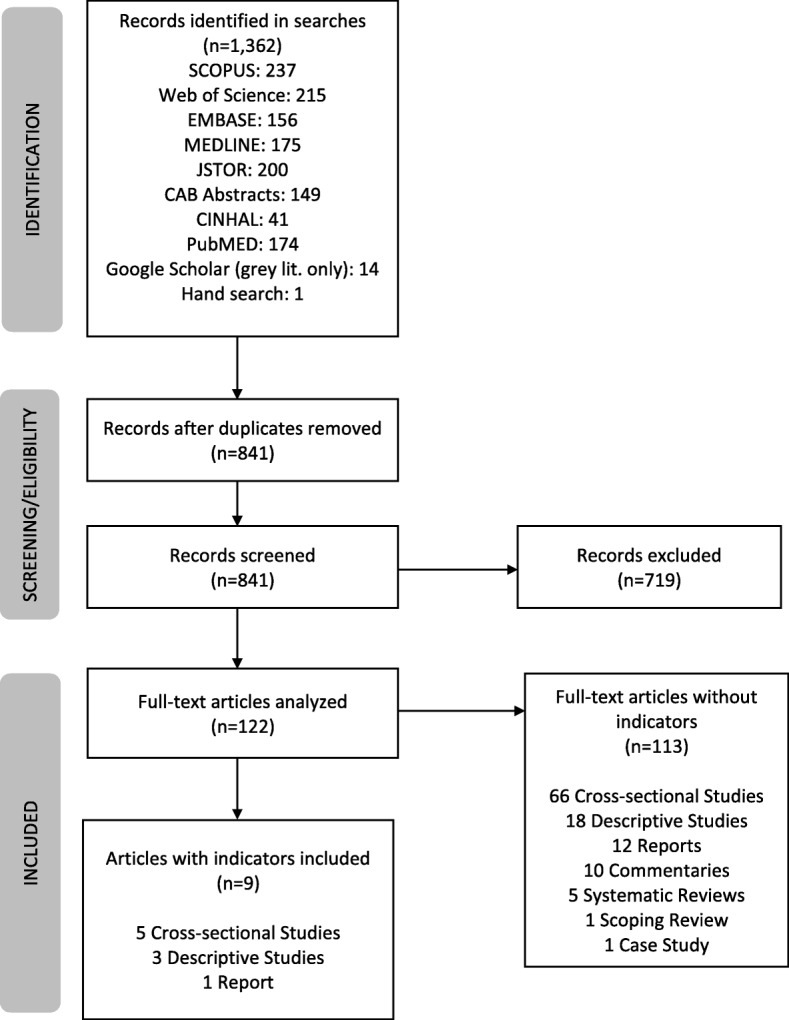
Flow diagram of search outcome

A systematic search of all available English language literature (all dates up to July 2018) was conducted using the following search terms: (teenager OR adolescent) AND “food marketing”; (teenager OR adolescent) AND “food advertising”. Databases used included Scopus, Web of Science, Embase, Medline, JSTOR, CAB Abstracts, CINHAL, and PubMed. Google Scholar was used to locate grey literature: the first 800 entries in the search return list were scanned, as per the recommended protocol for topic searches, which identifies saturation at page 80 [27]. A hand search was also conducted of the websites for the World Health Organization (WHO), the Federal Trade Commission (FTC), and the Institute of Medicine (IOM). Figure 1 outlines the search strategy.

### Inclusion criteria

All peer-reviewed journal articles, book chapters, and grey literature examining food marketing to youth as the central topic were included, along with any study in which any individual in the age 12–17 range appeared (whether labelled as ‘teenager’ or overlapping with/collapsed into ‘child’ or ‘adult’ categories). Included were all study types (including original research, reviews, commentaries and reports) and all food promotion platforms and techniques. Excluded were studies that examined whole foods (as opposed to packaged/processed food), alcohol advertising, parental opinions about food marketing to teenagers, and general screen time exposure (compared to screen time for food marketing). Also excluded were studies examining unhealthy products marketed to children where food is only one category of products (i.e., food, cigarettes, alcohol), as well as studies where food advertising is only one of multiple factors being examined as a determinant of health (e.g., alongside physical activity, nutritional value of foods, etc.). Finally, studies were excluded if they examined mass media effects on children’s attitudes and behaviours where food advertising is only one of multiple topics (e.g., alongside issues of body image or tobacco smoking).

### Data extraction and analysis

Abstracts were reviewed for selection by one author and two graduate students. The team extracted data for the following: study aims/objectives, study type, methods, population, food promotion platform and/or technique, country studied, and indicators of “teen-targeted” food promotion. Specifically, *population* was recorded in terms of both defined age range (i.e., 12–17) and/or label used for age group (i.e., teenager, adolescent, child, adult). Food promotion *platform* was defined as the communication channel, format or setting used to reach teenagers. Examples of *platforms* include television, social media, and convenience stores. Food promotion *techniques* was defined as the specific strategies or practices used to promote food brand or product awareness to teenagers. Examples of *techniques* include engagement tactics (i.e., viral marketing, info mining), and publicity tactics (i.e., philanthropic marketing, event sponsorship, etc.). *Indicators* refer to the criteria describing specific appeals in food marketing messages. Examples of *indicators* include characters or spokespersons, and themes. For the analysis, we reviewed and coded the following data categories: aims/objectives to isolate measured outcomes; population to identify variations in age ranges/labels used; food promotion platform and technique to categorize research focus; and indicators to identify common themes/categories in food marketing content.

## Results

We identified 841 abstracts, and marked 122 [6, 8, 10, 12, 16, 28–144] for inclusion in the broader study to define the scope of literature around food marketing to youth (subjects 17 and under). The identified literature dates from 1999 up until 2018, with the majority of studies appearing since 2013. Approximately half of the studies (52%) focus on the United States: this accounts for 63 of the 122 studies. The second and third ranked entries (Australia, *n* = 12; New Zealand, *n* = 10) represent only 10 and 8% of the total, respectively. The remaining studies come from the following countries/territories: Europe (*n* = 5), multiple countries worldwide (*n* = 5), Canada (*n* = 4), India (*n* = 3), South Korea (*n* = 3), United Kingdom (*n* = 3), China (*n* = 2), Chile (*n* = 1), Germany (*n* = 1), Italy (*n* = 1), Malaysia (*n* = 1), Malta (*n* = 1), Mexico (*n* = 1), Norway (*n* = 1), Poland (*n* = 1), Portugal (*n* = 1), Singapore (*n* = 1), Slovenia (*n* = 1), and Spain (*n* = 1).

### Populations identified

Studies focusing exclusively on teenagers (ages 12–17) represent only 18% of the literature identified. Instead, research more commonly examines child and teenage populations concurrently (36%), or includes teenager age ranges under the label of “child” (30%). With much less frequency, teenagers are examined concurrently with children and adults (7%), adults (5%), and as “children” alongside adults (3%).

### Outcomes and study approaches

The majority of *outcomes measured* belong to the category of advertising/media exposure (54%), with much smaller numbers of studies identified in relation to behaviours (13%), attitudes (11%), obesity (7%). Nineteen percent of the studies were reviews, reports and commentaries without measurable outcomes. In terms of *study type*, the majority of studies are cross-sectional (58%), while content analysis is the most frequently used *method* (46%).

### Marketing/promotions identified

Included studies described a variety of communication channels (or *platforms*) through which teen-targeted food marketing was delivered. These promotion *platforms* were grouped into three broad categories: 1) broadcast/mass media (i.e., television, radio, magazines, movies, sign/poster, billboards, food packaging); 2) digital (i.e., website, banner ads, smartphones, social media, video games, YouTube, mobile app); and 3) settings-based (i.e., schools, movie theatre, fast food restaurant, grocery store, convenience store, public transit, sports club). Broadcast/mass media accounted for 59% of platforms examined in existing studies, followed by digital at 25% and settings-based at 16%.

Numerous promotion *techniques* were described, and grouped into ten categories. Techniques included: 1) ad content/style (i.e., licensed/branded character, animation/sound effects); 2) brand familiarity (i.e., product placement, endorsers/influences); 3) digital engagement tactics (i.e., viral marketing, accounts/memberships and info mining); 4) events and publicity (i.e., philanthropic marketing, publicity); 5) games and play (i.e., advergames, quizzes/polls); 6) general engagement tactics (i.e., integrated or direct marketing, advertorials); 7) incentives (i.e., giveaways, coupons/rebates); 8) product elements (i.e., product design, appeals/claims); 9) settings-based tactics (i.e., retail/point-of-purchase displays, product sales in schools); and 10) sponsorship (i.e., celebrity endorsement, sports event sponsorship).

### Teen-specific marketing appeals

Of the 122 included studies, only 9 specified criteria, or *indicators*, to identify defining aspects of teen-targeted food marketing. Collectively, they offer a list of 42 indicators that describe the content of teen food advertising from characters and celebrities, to actors/models, to themes and activities. These studies and their indicators are reported in Table 1.

**Table 1:** Summary of studies reporting indicators to identify teen-targeted food marketing

Study	Teen Population	Indicators of teen-targeted food/beverage marketing
Federal Trade Commission (2008) [59]	12–17 year olds	-animated or licensed characters -popular celebrities -language of ‘teen/teenager/adolescent’ -teenaged performers/models/characters -teen themes/activities/incentives/products/media (Appendix B, attachment C: p. C3, C13)
Harris et al. (2010) [79]	17 and under (classified as “general audience”)	-product or personality from TV (general audience), PG-13 movie, or other type of entertainment, or sports, or product (p. 411)
Harris et al. (2011) [81]	12–17 year olds	-‘placed to reach teens’ - teenaged main characters (also targeting race/ethnicity) - addresses teen directly - promotes teen products - uses techniques appealing to teens (i.e., social media) (p. 114)
Potvin Kent et al. (2011) [113]	10–12 year olds	-‘fun’ (i.e. product has playful shape, colour, taste, and/or depiction of interactions with the product in ad show high levels of enjoyment) -media characters or celebrities (i.e., licensed characters, and sport, TV, movie, and music celebrities) (p. e435)
Interagency Working Group on Food Marketing to Children (2011) [85]	12–17 year olds	-animated or licensed characters -teen language -teenaged models -teen themes/activities/incentives -appeal to teens to participate in promotion (p. 19)
Federal Trade Commission (2012) [60]	12–17 year olds	-animated or licensed characters -popular celebrities -language of ‘teen/teenager/adolescent’ -teenaged performs/models/characters -teen themes/activities/incentives/products/media (Appendix B, attachment C: p. C3, C13)
Potvin Kent et al. (2014) [114]	12–17 year olds	-characters that teens identify with -adolescent activities (i.e., school dances, video games) -teen music -adolescent themes (i.e., popularity, freedom) -teen humour (p. 2055)
Théodore et al. (2017) [133]	15–18 year olds	-adolescent characters -adolescent context/themes (i.e., high schools, concerts) (p. 314)
Vandevijvere et al. (2017) [137]	13–17 year olds	-adolescent themes (i.e., fashion, image, sexuality) (p. 34)

**Fig. 2:**
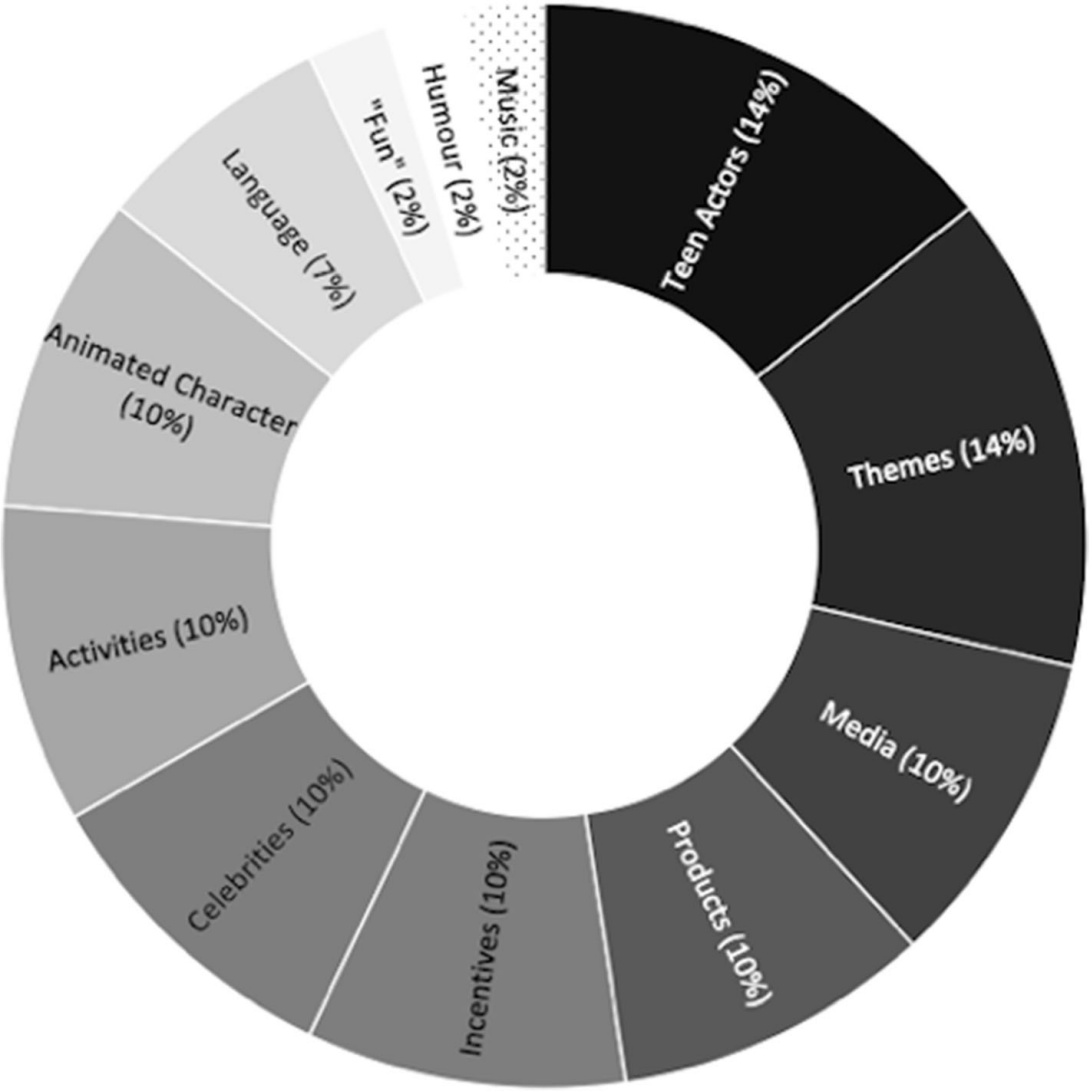
Indicators of teen-targeted food marketing, by frequency across nine studies

The indicators reported in Table 1 were then summarized into 12 categories to represent different aspects of marketing content targeting teens. These indicator categories are: *teen actors, teen themes, teen media, teen products, teen incentives, celebrities that appeal to teens, teen activities, animated characters, teen language, “fun”, teen humour,* and *teen music.* The reported frequencies of these categories across the studies identified are represented in Fig. 2. As illustrated, a wide variety of indicators are in use across a small number of studies (7%), while the vast majority of literature uses no indicators at all to identify teen-specific food marketing content (93%).

## Discussion

This study highlights that there are very few food marketing studies that focus exclusively on teenage populations, despite this group being highly targeted and vulnerable to promotional appeals [1, 7, 11]. The problem with examining teens alongside, or as part of the larger group of, child populations is that teen-specific marketing appeals are rarely differentiated from child-focused ones. This presents challenges in terms of identifying and accurately measuring the presence of teen food promotion. Indeed, in existing literature, such content is often passively identified by selecting a promotional platform that targets teenagers (e.g., a television channel), and then performing a content analysis of its advertisements. In such studies, the main outcome being measured is *exposure level* to food marketing (i.e., number of commercials within a given time frame), with limited attention to the actual *content* of those messages. The assumption is that, based on the platform, the advertisements will by default appeal to teenagers.

As this scoping review reveals, television advertising remains the primary focus of literature on youth food marketing: 40% of the included studies focus exclusively on television programming, and 66% overall include television as an object of study (as the main focus, or in addition to other platforms). This emphasis on television in existing food marketing studies on youth means that other platforms (e.g.., digital, settings-based), which represent important sources of teen-targeted messaging, are less understood. Indeed, the World Health Organization has identified regulatory focus on broadcast advertising as a key challenge to implementing policy on food marketing to young people because it ignores the digital sphere (as well as sponsorship and product packaging) [7]. Settings-based marketing also requires more attention: a recent scoping review found that exposure levels for children under age 17 in Canada are potentially underestimated, as they are marketed to in their homes, schools, and supermarkets [10].

The existing focus on television programming also excludes the range of promotion techniques now used to market food and beverages to youth [3]. We define promotion *techniques* as specific strategies used to promote brand awareness to youth, as opposed to promotion *platforms* which are the communication channels used to reach them. In the literature identified, promotion *platforms* and *techniques* were often conflated. This is problematic because the former is simply a delivery mechanism, while the latter is a targeted strategy or practice. Stated differently, food marketing platforms can be used to measure exposure to advertising, but the power of that advertising is more accurately assessed by examining the content of promotion techniques [145].

This study identified a remarkable variety of promotion techniques in use to target young people (17 and under): 78 unique items were grouped into the ten categories (described in the results section), ranging from settings-based and digital tactics, to more traditional approaches such as brand familiarity and sponsorship/endorsements. When it comes to teen food marketing, promotion techniques are significant as teenagers may be targeted by specialized techniques that do not reach younger children. For example, consider *information gathering/info mining* or *incentives* that are the result of signing up for subscriptions or accounts for registered users over the age of 13; *point-of-purchase displays* in stores where teenagers use their own spending money; *viral marketing and/or word-of-mouth marketing* (which may be significant due to the importance of peer acceptance), and the social/symbolic value of food (brands, or ‘cool’ food products) within the peer group [146, 147]. However, the *content* of these targeted messages, not simply the type of strategy, matters as well. It is critical to identify marketing content that appeals to teens in order to better understand its potential impacts. For instance, when it comes to “special offer” promotions, are teens incentivized by coupons or by limited-edition products? Which tactic is more likely to influence their food preferences and/or motivate their purchase behaviours?

The need for improved understanding of the content of teen-targeted food marketing is particularly acute in the Canadian context, where Health Canada is preparing to implement Bill S-228, the Child Health Protection Act, which will prohibit the marketing of unhealthy food and beverages to children under age 13 [26]. As part of this initiative, Health Canada has committed to monitor the marketing of unhealthy foods to teenagers aged 13–17, in order to assess whether food and beverage marketing to teenagers will increase post-legislation. Additionally, the World Health Organization has identified the need for worldwide monitoring of digital marketing to children under 17, in light of the increasing popularity of social media/mobile devices for this age group which involves targeted/personalized advertising [148]. Monitoring depends not only upon the ability to accurately identify *where* and *when* food marketing reaches teens, but also *how* it appeals to them, and the use of indicators is critical to this process. The indicators identified in this study are a good starting point for considering how teen-targeted marketing appeals are specific to that age group. However, some indicators are clearer than others for determining inclusion/exclusion. For example, the presence of a *teenaged actor* or *animated character* counts as teen-targeted, as does *language* that directly addresses the teen audience (i.e., the terms “teen”, “teenager”, “adolescent”). These are straightforward to identify. The following categories are less clear: *teen theme, teen activity, celebrities that appeal to teens, teen incentives, teen humour*, and *teen music*. Specific definitions are needed for each of these categories so that the interpretation of these terms is not reliant on the subjective opinions of researchers or policy makers. Finally, several of the indicators identified in existing literature are completely unclear as inclusion criteria: does the category of “*media*” refer to the platform used to reach teens, or the use of teen media as a theme (i.e., image of a smartphone in the advertising)?; does “*product*” refer to ones made-for-teens, or to any products that teens find appealing?; and where the indicator “*fun*” refers to a product and its representation in advertisements, is this characteristic valued by teen audiences, or are they perhaps more concerned with “cool/trendy”? Additional qualitative research is needed to determine how teens themselves describe and define food marketing directed at them, in order to properly differentiate between teen-specific, child, and adult oriented content, and to inform conceptions of teen indicators.

It is important to note a limitation of this review: the summary of indicators is based on a small number of identified studies, and their validity needs to be tested in future research. Studies that focus exclusively on the 12–17 year old age range are needed in order to examine the uniqueness of teen appeals. Nuanced research in this area will provide much needed insight into the power of teenager-targeted food marketing, and its potential impacts on food attitudes and behaviours.

## Conclusions

Food and beverage marketing to teenagers is ubiquitous. However, little focused research exists on teenage populations, despite their vulnerability. The most commonly measured outcome in studies of food marketing to young people (17 and under) is exposure to advertising—and most often in television programming—which ignores the increasing range of promotion platforms and techniques specifically targeting teenagers. More research is needed to identify teen-specific food marketing content, and this scoping review is the first to analyze the specific indicators currently being used for evaluating such appeals. The ability to isolate teen-targeted food and beverage marketing will allow for more effective monitoring of its presence, and assessment of its power and potential impacts on teenagers, in order to inform policy around its regulation.

## Data Availability

Not applicable
